# Evaluating DNA Methylation, Gene Expression, Somatic Mutation, and Their Combinations in Inferring Tumor Tissue-of-Origin

**DOI:** 10.3389/fcell.2021.619330

**Published:** 2021-05-03

**Authors:** Haiyan Liu, Chun Qiu, Bo Wang, Pingping Bing, Geng Tian, Xueliang Zhang, Jun Ma, Bingsheng He, Jialiang Yang

**Affiliations:** ^1^Academician Workstation, Changsha Medical University, Changsha, China; ^2^College of Information Engineering, Changsha Medical University, Changsha, China; ^3^Department of Oncology, Hainan General Hospital, Haikou, China; ^4^Geneis Beijing Co., Ltd., Beijing, China; ^5^Qingdao Geneis Institute of Big Data Mining and Precision Medicine, Qingdao, China; ^6^Department of Oncology, Jiamusi Cancer Hospital, Jiamusi, China

**Keywords:** tumor tissue-of-origin, DNA methylation, gene expression, somatic mutation, multi-classifier XGBoost, pearson correlation algorithm

## Abstract

Carcinoma of unknown primary (CUP) is a type of metastatic cancer, the primary tumor site of which cannot be identified. CUP occupies approximately 5% of cancer incidences in the United States with usually unfavorable prognosis, making it a big threat to public health. Traditional methods to identify the tissue-of-origin (TOO) of CUP like immunohistochemistry can only deal with around 20% CUP patients. In recent years, more and more studies suggest that it is promising to solve the problem by integrating machine learning techniques with big biomedical data involving multiple types of biomarkers including epigenetic, genetic, and gene expression profiles, such as DNA methylation. Different biomarkers play different roles in cancer research; for example, genomic mutations in a patient’s tumor could lead to specific anticancer drugs for treatment; DNA methylation and copy number variation could reveal tumor tissue of origin and molecular classification. However, there is no systematic comparison on which biomarker is better at identifying the cancer type and site of origin. In addition, it might also be possible to further improve the inference accuracy by integrating multiple types of biomarkers. In this study, we used primary tumor data rather than metastatic tumor data. Although the use of primary tumors may lead to some biases in our classification model, their tumor-of-origins are known. In addition, previous studies have suggested that the CUP prediction model built from primary tumors could efficiently predict TOO of metastatic cancers (Lal et al., 2013; Brachtel et al., 2016). We systematically compared the performances of three types of biomarkers including DNA methylation, gene expression profile, and somatic mutation as well as their combinations in inferring the TOO of CUP patients. First, we downloaded the gene expression profile, somatic mutation and DNA methylation data of 7,224 tumor samples across 21 common cancer types from the cancer genome atlas (TCGA) and generated seven different feature matrices through various combinations. Second, we performed feature selection by the Pearson correlation method. The selected features for each matrix were used to build up an XGBoost multi-label classification model to infer cancer TOO, an algorithm proven to be effective in a few previous studies. The performance of each biomarker and combination was compared by the 10-fold cross-validation process. Our results showed that the TOO tracing accuracy using gene expression profile was the highest, followed by DNA methylation, while somatic mutation performed the worst. Meanwhile, we found that simply combining multiple biomarkers does not have much effect in improving prediction accuracy.

## Introduction

Carcinoma of unknown primary (CUP) is a type of metastatic carcinoma whose primary tumor site is unknown. CUP accounts for approximately 3–5% of all human malignancies (Shaw et al., 2007; Conway et al., 2019; Xu et al., 2019). Since the treatment cannot be determined based on primary tumor site, CUP patients usually have poor prognosis. The median survival time of a CUP patient is 6–16 months even when empiric combination chemotherapy is employed (Pavlidis and Fizazi, 2005; Pentheroudakis et al., 2011; Jeyaram et al., 2019).

In order to improve the treatment of CUP patients and prolong their survival time, an accurate identification of tumor tissue-of-origin (TOO) is essential. Currently, there is a clinical practice for tracing the tissue origin of CUP, which involves physical examination, laboratory testing, immunohistochemistry, pathological imaging, and endoscopic examination. However, the results could be highly subjective and only the TOO of about 20–30% CUP patients could be revealed (Horlings et al., 2008; Bender and Erlander, 2009). For the past few years, molecular profiling of tissue-specific genes had become a promising technique for TOO tracing, due to its good diagnostic accuracy on poorly differentiated or undifferentiated tumors (Oien and Dennis, 2012).

With the increasing availability of high-throughput genomic and transcriptional data, there are several molecular biomarkers in The Cancer Genome Atlas (TCGA) including somatic mutation, copy number variation (CNV), gene expression, microRNA expression, and DNA methylation, which were used to trace cancer TOO (Li et al., 2017; Tang et al., 2018). The most popular biomarker used in TOO inference is gene expression. For example, Ma et al. (2006) demonstrated an overall success rate of 87% by using a 92-gene RT-PCR assay to identify the tissue origin of 32 different tumor types. Xu et al. (2016) identified a 154-gene expression signature that could discriminate the origin of 22 common human tumor types with an overall accuracy of 92%. DNA somatic mutation and CNVs are also frequently used to infer TOO. For instance, genomic profiling revealed an IDH1 somatic mutation, supporting the diagnosis of cholangiocarcinoma in a malignancy of unknown origin (Sheffield et al., 2016). In some reports, the tumor-specific enrichment for mutations in certain genes (sometimes mutations at specific locations within genes) had also been observed and used to infer tumor location (Dietlein and Eschner, 2014; Lawrence et al., 2014). Based on this observation, mutation burden in genes were used to infer tumor TOO; however, the performances are usually not very well (He et al., 2020; Liu et al., 2020). In addition, Küsters-Vandevelde et al. found that particular CNVs may be associated with cancer metastasis (Küsters-Vandevelde et al., 2017; Zhu et al., 2019). As such, Liang et al. compared several computational methods using CNV features and achieved good performances in inferring TOO for six cancer types (Liang et al., 2020).

Finally, there are also a few methods to trace tumor TOO by integrating multiple biomarkers. For example, Hoadley et al. proposed a method to trace the origin of 12 cancer types based on methylation and CNV (Hoadley et al., 2014; Zhang et al., 2019). Marquard et al. (2015) applied both point mutations and copy number aberrations (PM + CN) classifiers to obtain a classification accuracy of 85% across six primary cancers. He et al. (2020) combined molecular data of somatic mutation and gene expression profiling to infer cancer TOO and achieved a 10-fold cross-validation prediction accuracy of around 96% using the random forest classification method across 20 solid tumors.

Although many previous studies have used molecular profiles such as DNA methylation, somatic mutation, gene expression, and their combinations to predict the tissue origin of CUP, there is still no systematic comparison among them to our best knowledge. In addition, the epigenetic studies on CUP are more or less ignored. To address this need, we aim to compare the predictive ability of these biomarkers and combinations in a unified background. In addition, we aim to investigate whether multi-biomarkers can significantly improve prediction accuracy compared to single biomarkers.

## Materials and Methods

### Data Preparation

The publicly available datasets for gene expression profile (assembly_version: GRCh37, platform: Illumina HiSeq, experimental_protocol: RNASeqV2_RSEM_genes^1^), somatic mutation (assembly_version: GRCh37, platform: Illumina GA sequencing, variation_calling_algorithm:TCGA-MC3^2^), and 450 k DNA methylation array data (HumanMethylation450_after_2011_08_02) of the 21 different tumor types were collected from the ICGC data portal^3^. We used samples from 21 primary tumors as training and validation datasets to construct and validate models for inferring CUP. The data from raw TSV files were pre-processed by extracting and deduplicating, respectively, generating three feature matrices with “*p*” rows of the tumor samples numbers and “*q*” columns of gene numbers across aforementioned three categories of biomarkers. Each sample with histologically confirmed origins was tagged for its type of cancer. In particular, the somatic mutation data was extracted and deduplicated to form a feature matrix according to information of icgc_donor_id, chromosome, chromosome_start, and gene_affected before the feature value divided by the length of the gene. In total, 7,224 TCGA samples originating from 21 cancer types were downloaded in our work. Detailed information on the number of samples of each cancer type can be found in Table 1.

**TABLE 1:** Sample information of each cancer from TCGA database.

**Available cancer types**	**Abbreviation**	**Samples**	
		**Amount**	**Percentage**
Bladder urothelial carcinoma	BLCA	271	3.75%
Breast invasive carcinoma	BRCA	942	13.04%
Cervical squamous cell carcinoma and endocervical adenocarcinoma	CESC	225	3.11%
Colon adenocarcinoma	COAD	383	5.30%
Glioblastoma multiforme	GBM	131	1.81%
Head and neck squamous cell carcinoma	HNSC	461	6.38%
Kidney renal clear cell carcinoma	KIRC	338	4.68%
Kidney renal papillary cell carcinoma	KIRP	211	2.92%
Acute myeloid leukemia	LAML	119	1.65%
Brain lower grade glioma	LGG	433	5.99%
Liver hepatocellular carcinoma	LIHC	227	3.14%
Lung adenocarcinoma	LUAD	472	6.54%
Lung squamous cell carcinoma	LUSC	407	5.64%
Ovarian serous cystadenocarcinoma	OV	186	2.57%
Pancreatic adenocarcinoma	PAAD	111	1.54%
Prostate adenocarcinoma	PRAD	352	4.87%
Rectum adenocarcinoma	READ	137	1.90%
Skin cutaneous melanoma	SKCM	423	5.86%
Stomach adenocarcinoma	STAD	415	5.74%
Thyroid carcinoma	THCA	486	6.73%
Uterine corpus endometrial carcinoma	UCEC	494	6.84%
Total		7,224	100%

### Data Combination

We combined the feature matrix of gene expression, somatic mutation, and DNA methylation, respectively, and generated seven different feature matrices, including a 7,224 × 20,501 gene expression feature matrix, a 7,224 × 34,618 somatic mutation feature matrix, a 7,224 × 13,869 DNA methylation feature matrix, a 7,224 × 55,119 both gene expression and somatic mutation feature matrix, a 7,224 × 34,370 both gene expression and DNA methylation feature matrix, a 7,224 × 48,487 both DNA methylation and somatic mutation feature matrix, as well as a 7,224 × 68,988 the feature matrix that combines these three biomarkers. Then, only the samples shared in the seven feature matrices were selected for a fair comparison. In addition, we performed the L1 normalization on the columns of each feature matrix such that each entry was divided by the sum of the corresponding column. So, the samples data of 7,224 tumor samples in these 21 different tumor types after filtering data and normalizing each feature matrix are obtained.

### Gene Feature Identification

In order to minimize the number of genes while maintaining the highest primary tracing accuracy possible, we employed Pearson correlation algorithm as the feature selection method. According to the mechanism of feature selection, we screened out the sets of genes by Pearson correlation algorithm (Hall, 1998; Saeys et al., 2007) using one-vs-all method where one cancer was used as positive and the other cancer types were together used as negative. Next, the selected genes were ranked in descending order according to their importance, with the most informative ones appearing at the top of the list. We identified the top N genes from each cancer type and merged into a list after removing the redundant ones, and then we further used all of the identified genes to classify each sample among all the TCGA samples separately for internal cross-validation.

### Multi-Classifier XGBoost

XGBoost (Extreme Gradient Boosting) was a learning framework based on boosting tree models for solving supervised learning problems. In this study, all genes obtained from the above step were used to train the classification model based on XGBoost because of its excellent scalability and operation (Ji et al., 2019; Lv et al., 2020; Yu et al., 2020). XGBoost performed a second-order Taylor expansion on the loss function and it could automatically use the CPU’s multithreading for parallel computing. We first used bootstrap method to generate *k* training sets and then each train set that consists of a set of samples was used to construct a tree. After XGBoost mapped each sample to its corresponding leaf node, its final predicted value was the sum of the corresponding leaf node values for each tree. To control the complexity of the model and prevent overfitting, the L2 regularization term was applied and the maximum depth was set to three. Normally, we could not enumerate all possible tree structures and pick the best, so we chose a greedy algorithm instead: we started with a single leaf and iterated and split to add nodes to the tree. When splitting a node, in order to restrain the growth of the tree and help avoid overfitting of the model, a splitting threshold for information gain was added. The leaf node was allowed to split if and only if the information gain is greater than the splitting threshold. In addition, for obtaining relatively stable and reliable results, possibly minimizing the percentage of false positives and false negatives, 10 times 10-fold cross-validation based on the whole dataset was used. The XGBoost method for classification had proper separation of training and test data during features selection; in other words, feature selection is performed from the training set once in each 10-fold cross-validation.

## Results

### Datasets Used in This Study

**FIGURE 1:**
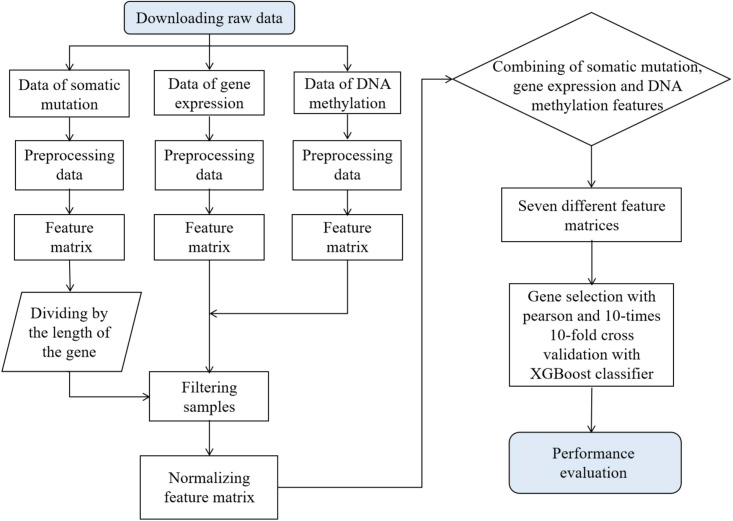
Flow diagram of prediction on cancer tissue origin and performance evaluation. Seven different feature matrices, respectively, are gene expression feature matrix, somatic mutation feature matrix, DNA methylation feature matrix, both gene expression and somatic mutation feature matrix, both gene expression and DNA methylation feature matrix, both DNA methylation and somatic mutation feature matrix, and the feature matrix that combines these three biomarkers.

To compare the accuracy and robustness of different biomarkers in terms of cancer type prediction, publicly available gene expression profile, somatic mutation, and DNA methylation data from 7,224 samples were collected from TCGA for this study. The complete workflow is shown in Figure 1. We first download the original data from the TCGA database and generate the feature matrix after pre-processing such as extraction and de-duplication, respectively. Second, 7,224 tumor samples were left after the sample filtration. Third, the generated feature matrix underwent the normalization treatment. Table 1 shows sample information for each cancer, and we found that each of the 21 cancer types had a sample size of more than 100, while the largest sample size was breast cancer (942 samples) and the smallest was pancreatic cancer (111 samples).

### A General Framework of This Study

Due to the intra-tumor heterogeneity, it was critical to identify the most informative genes from the high-dimensional datasets in order to better distinguish true mutation from background noise. Pearson correlation-based feature selection was characterized by fastness in operation speed and simple in complex calculation, which made it a successful multi-variable filtering method for high-dimensional data analysis. It was used to assess correlations between cancer types and corresponding gene features. Hence, we adopted Pearson correlation to select the most informative genes from the generated seven different feature matrices for classification detection. This process consisted of the following four steps: First, we created an array and binarized each row and column of 7,224 tumor samples. If the samples belonged to the tumor type, they would be labeled as “true;” otherwise, they would be labeled as “false.” Second, we calculated the correlation of the feature with samples labeled “true” for each cancer type and then sorted in decreasing order according to their correlation. Third, we took the most important signature, which appeared in the first N genes of the list for each cancer type, where N was an integer. Fourth, we combined the first N genes in lists of 21 cancer genes and removed the redundant genes. Using a series of integers, we generated a corresponding number of gene sets for further classification.

### Gene Expression Profile Outperforms Other Biomarkers and Combinations in Inferring Tumor TOO

**FIGURE 2:**
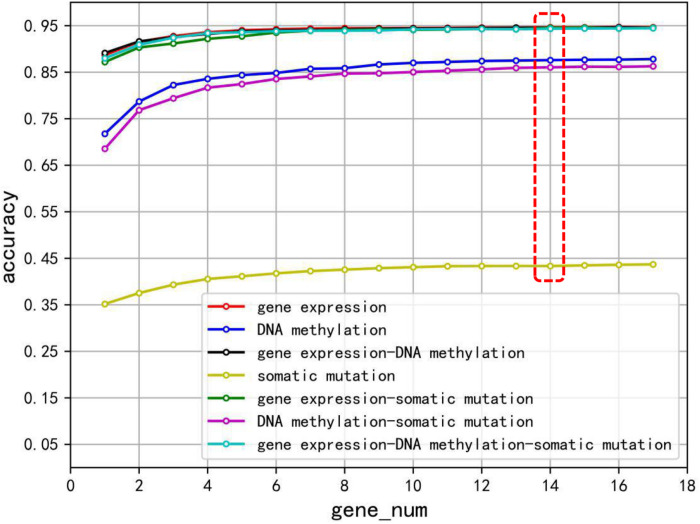
The classification accuracy of using gene expression, somatic mutation, DNA methylation, and combination of the three biomarkers, respectively, on each gene set.

To evaluate the performance of the biomarker genes of gene expression profiling, somatic mutation, DNA methylation, and different combinations of them, a 10-fold cross-validation method was used to train XGBoost classification model. Especially to avoid overfitting of XGBoost algorithm, we achieved relatively stable and reliable results through 10 times 10-fold cross-validation, and minimized the percentage of false positives and false negatives as much as possible. The accuracies are shown in Figure 2. Different gene sets were used for cross-validation, and seven different polylines representing the accuracy of each 10 times 10-fold cross-validation were plotted. Clearly, using too few genes did not achieve the desired classification effect, until a list was used that combined the list of the 14 top-ranked genes for each cancer type and removed redundant genes. Although a gene set with more genes can achieve better accuracy, the growth was slow. The best classification performance was given by using data of gene expression (the mean accuracy was 94.63%), while the worst classification effect was obtained by using somatic mutation data (the mean accuracy was 43.33%), and other biomarker combinations were in the middle level.

**FIGURE 3:**
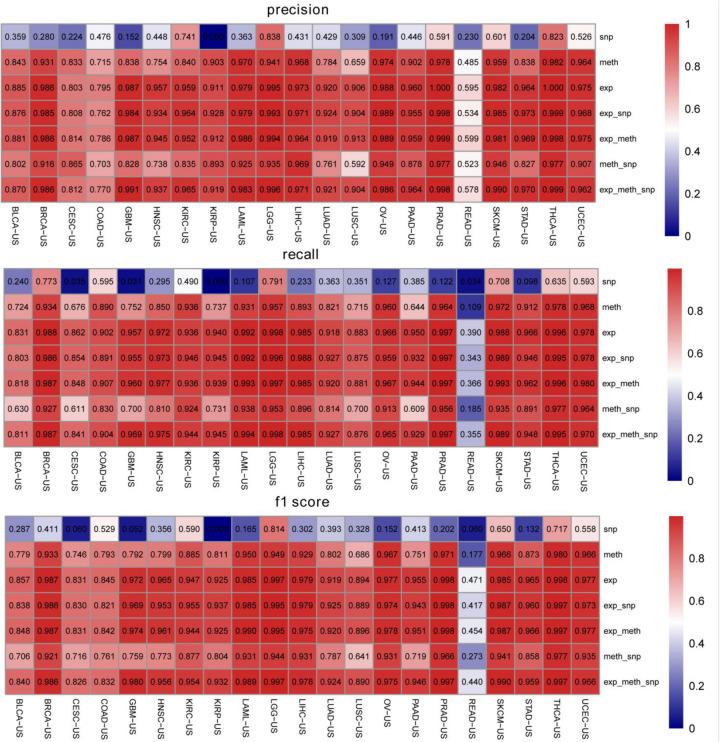
The classification precisions, recall rates, and f1 scores for each biomarker combination on the 14 top-ranked genes for each cancer type. exp represents gene expression profiling, meth represents DNA methylation, and snp represents somatic mutation.

We compared the classification performance of various combinations of biomarkers to get through the evaluation index of recall rate, precision, and f1 score. We plotted the heat map of mean value of recall, precision, and f1 score on the 14 top-ranked genes for each cancer type. In Figure 3, the rows represented the cancers and columns denoted the seven combinations of biomarkers. The gene expression classification performance was the best and the somatic mutation was the worst, which were consistent with the previous results in Figure 2. Figure 3 shows that the combination of multiple biomarkers did not necessarily achieve higher classification accuracy.

**FIGURE 4:**
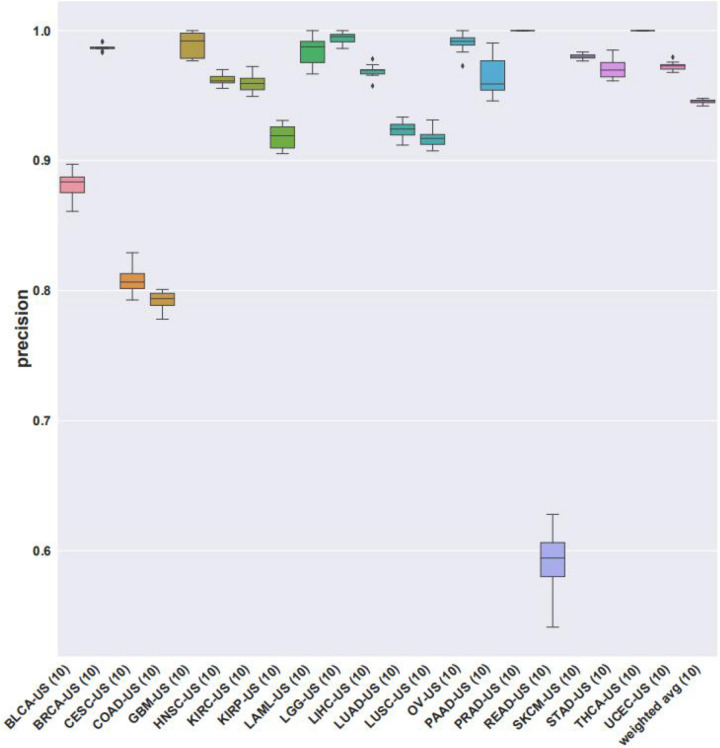
The precisions of XGBoost classifier using gene expression data on the 14 top-ranked genes for each cancer type. Precisions from 10 times of cross-validations were averaged.

**FIGURE 5:**
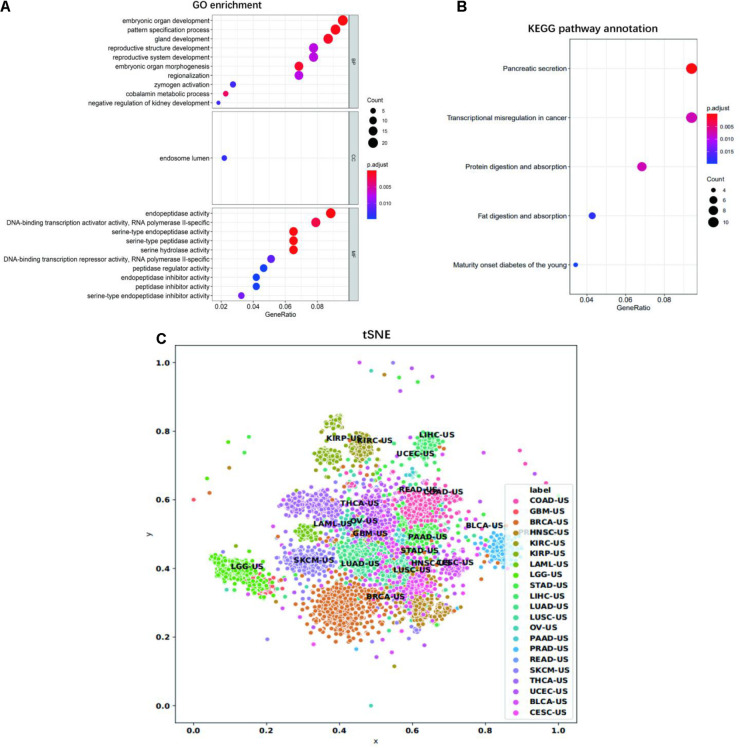
GO and KEGG analysis. **(A)** Significantly enriched GO cellular component, biological process, and molecular function of selected 14 top-ranked genes of each cancer type in gene expression data. **(B)** Significantly enriched KEGG pathways of the selected 14 top-ranked genes of each cancer type in gene expression data. The dot plot shows the number of signature genes identified by enrichment analysis for each cell component, biological process, molecular function, and KEGG pathway. The dot size represents the number of genes enriched in specific pathways and the dot color represents adjusted enrichment *p*-value. **(C)** The tSNE visualization of all samples for the 21 tumor types. The *x*- and *y*-axis represent the first and second dimension of tSNE, respectively.

We then looked at the performance of XGBoost algorithm using only gene expression values as the train features and fitting the cancer type as labels. We used 10 times of the 10-fold cross-validation method to evaluate the classification performance of each cancer on the 14 top-ranked genes. In two cancer types (PRAD and THCA), the precision was 100%. However, the precision obtained by READ and COAD was lower, at 79.20 and 59.15%, respectively. The precisions corresponding to each cancer were plotted as Figure 4. Gene ontology (GO) enrichment analysis was performed to study the selected signature genes in cellular component, biological process, and molecular function. Figure 5A shows that the most biological significance related to the 14 top-ranked genes of each cancer type in gene expression data by GO analysis was biological processes and molecular functions. Kyoto Encyclopedia of Genes and Genomes (KEGG) pathway analysis was also used to understand the target genes from gene expression. Figure 5B shows the most enriched KEGG pathways. For the visualization of samples from 21 tumor types, we performed cluster analysis as represented by t-distributed stochastic neighbor embedding (t-SNE) plots in Figure 5C. Samples from the 21 cancer types could be roughly distinguished.

## Discussion

Data of gene expression profiling, somatic mutation, and DNA methylation can be used to identify the primary site of tumors. However, for the first time, the three biomarkers and their combinations have been used to identify the origin of tumor tissues, and their ability to trace the origin of primary tumors has been compared and analyzed. We carried out a large number of experiments by using a shared sample of 7,224 and combined data from 21 cancer types. By comparing their performance, we found that the gene expression profile data obtained the highest accuracy, while the combined data could not obtain better classification performance. The comparison results are shown in Figure 2. While it was difficult to know exactly what led to some misclassification in combined datasets, the batch effects of RNA-seq and methylation data may have had a negative effect to our results.

XGBoost has been proven to have better performance than other more traditional models in many machine learning tasks, so we used the XGBoost algorithm to construct the classification model and tested it on seven different biomarker combinations. The results showed that the XGBoost algorithms can predict the cancer type of unknown primary tissue with an efficient accuracy. We chose the 14 top-ranked genes from each cancer and put them together for classification. Our results indicated that the gene expression data obtained an accuracy of 94.63%, which is the highest prediction accuracy. However, the prediction accuracy of the combination with gene expression data with other data was slightly lower than that of using gene expression. The same thing happened with DNA methylation data, which alone had a slightly higher prediction accuracy of 87.59% than both data of DNA methylation and somatic mutation on prediction of cancer tissue origin. Somatic mutation had the worst classification of cancers with a terrible prediction accuracy of 43.33%.

Due to the optimal classification performance of gene expression profile, we further functionally annotate the union of 14 top-ranked genes of each cancer type in the gene expression data. The enrichment results are shown in Figure 5. GO analysis showed that the selected genes mainly participated in embryonic organ development/morphogenesis, pattern specification process/regionalization, gland development, reproductive system/structure development, DNA-binding transcription activator/repressor activity, RNA polymerase II-specific, serine-type endopeptidase/peptidase activity, and endopeptidase/peptidase inhibitor activity. In the KEGG pathway analysis, the top two significantly enriched pathways were “Pancreatic secretion” and “Transcriptional misregulation in cancer.” Other significant pathways included “Protein digestion and absorption,” “Fat digestion and absorption,” and “Maturity onset diabetes of the young.” Our signature genes were involved in these pathways, which might be useful in inferring cancer TOO. For example, ABCC1 is highly expressed in lung cancer tissues. ALX1 plays a key role in tumor progression and metastasis, and it has been shown to regulate the expression of genes that induce epithelial to mesenchymal transition in primary mesenchymal cells (Wu et al., 2008; Yuan et al., 2013; Yang et al., 2015; Yao et al., 2015). Remarkably, we found that KLK4 is ectopically expressed in human colon cancer and ovarian cancer cells, which is one of the members of the cancer-related KLK family (Walker et al., 2014; Loessner et al., 2018). GATA3 is an important transcription factor to regulate cell differentiation. GATA3 is up-regulated in ulcerative colitis (Christophi et al., 2012; Alhassan Mohammed et al., 2018), which is associated with increased risk of colorectal cancer (Gupta et al., 2007). Genes involved in these biological processes and KEGG pathways play a role in distinguishing between different types of cancer.

In our study, all data came from the TCGA dataset, and the batch effects of RNA-seq and methylation data may have had a negative effect to our results. However, it is unclear whether the batch correction methods will bring some additional bias and which batch correction method is correct. In TCGA, each sample was divided normalized such that the total number of transcripts is 1,000,000, which actually performs a very rough batch correction. Finally, we added Figure 5C, which suggests that the samples from different tumors could be roughly separated. This indicates that the batch effects might not dominate the results.

There are some limitations to our study. First, we constructed and assessed the models based on TCGA primary tumor data rather than metastatic tumor data, because it is extremely difficult to collect metastatic samples with a known primary tumor site. In the future, we will try to collect metastatic cancer samples to construct CUP prediction models or test known models. Second, we did not supply an independent dataset for validation since we could not find a database other than TCGA, which has data on gene expression, DNA methylation, and somatic mutation simultaneously. Finally, we only simply concatenated the features of different biomarkers. It might be better to test the effects of interaction terms since the biomarkers are not independent biologically.

In summary, this is the first study to compare the power of different biomarkers in inferring cancer TOO under the same condition, including the same dataset, the same preprocessing scheme, and the same classification algorithm. In the future, we will try to include metastasis tumor samples into our study, incorporate independent testing samples, and add interaction terms and novel classification models for improving prediction accuracy.

## Conclusion

The identification of the origin of tumor tissue was a challenging task. With a large number of molecular profiling, we can use them alone or combine some of them to improve the identification of primary tumor sites. Although we used primary tumor data, the primary information they provided were the most important to pinpoint the exact TOO for CUP. Machine learning algorithms were also effective tools to help classify cancers. The number of features used can greatly affect predictive performance. In this study, we used gene expression profiles, somatic mutation, and DNA methylation data to generate the feature matrix. Then, the optimal number of genes was obtained according to Pearson correlation algorithm, and the classification model was established using XGBoost algorithm. The same approach was used to compare the performance among a combination of some of the aforementioned biomarkers. The experimental results showed that the highest accuracy can be achieved by using gene expression profiling, but combining multiple biomarkers could not achieve better prediction performance.

## Data Availability Statement

The original contributions presented in the study are included in the article/supplementary material, further inquiries can be directed to the corresponding author/s.

## Author Contributions

JY and BH designed the study. HL, CQ, BW, and PB collected the data, analyzed the data, interpreted the data, and wrote the manuscript. HL, GT, and JM performed the experiment. GT, XZ, and JM reviewed the manuscript. All authors contributed to the article and approved the submitted version.

## Conflict of Interest

BW, GT, and JY were employed by the company Genesis Beijing Co., Ltd. The remaining authors declare that the research was conducted in the absence of any commercial or financial relationships that could be construed as a potential conflict of interest.

## Footnotes

1 https://tcga-data.nci.nih.gov/tcgafiles/ftp_auth/distro_ftpusers/anonymous/tumor

2 https://gdc.cancer.gov/about-data/publications/mc3-2017

3 https://dcc.icgc.org/releases/release_28/Projects
